# Comparison of the Effectiveness and Safety of d-Penicillamine and Zinc Salt Treatment for Symptomatic Wilson Disease: A Systematic Review and Meta‐Analysis

**DOI:** 10.3389/fphar.2022.847436

**Published:** 2022-03-18

**Authors:** Shan Tang, Li Bai, Wei Hou, Zhongjie Hu, Xinyue Chen, Jing Zhao, Chen Liang, Wei Zhang, Zhongping Duan, Sujun Zheng

**Affiliations:** ^1^ The First Unit, Department of Hepatology, Beijing YouAn Hospital, Capital Medical University, Beijing, China; ^2^ The Fourth Unit, Department of Hepatology, Beijing YouAn Hospital, Capital Medical University, Beijing, China; ^3^ Beijing Key Laboratory of Liver Failure and Artificial Liver Treatment Research, Beijing, China

**Keywords:** Wilson disease, symptomatic, pharmacological therapy, meta-analysis, systematic review

## Abstract

**Background:** Pharmacological therapy is currently the main treatment method for patients with Wilson disease (WD). We aimed to evaluate the efficacy and safety of the common treatment regimens in these patients.

**Methods:** We conducted a systemic review and meta-analysis by searching multiple databases for studies from inception to October 2021. Outcomes of interest were the improved rate and safety of d-penicillamine and zinc salts treatment in WD patients. Two independent reviewers performed the study selection and data extraction.

**Results:** Sixteen studies were included in this meta-analysis. The pooled improved rate for all included symptomatic WD patients was 78.0% (95% CI: 70.8%–85.2%). In symptomatic hepatic WD patients, there is no difference in the treatment efficiency of d-penicillamine and zinc salts (RR: 0.98, 95% CI: 0.86%–1.12%; *p* = 0.765). In neurological WD patients, the pooled improved rate of those who received d-penicillamine and zinc salts was 56.3% (95% CI: 37.5%–75.1%) and 80.2% (95% CI: 67.2%–93.2%), respectively. The incidence of adverse effects (RR: 2.42, 95% CI: 1.20%–4.88%; *p* = 0.014) and neurological deterioration (RR: 1.96, 95% CI: 1.31%–2.93%; *p* = 0.001) in all symptomatic WD patients treated with d-penicillamine was both higher than that of patients treated with zinc salts.

**Conclusion:** Our analysis suggests that symptomatic WD patients treated with d-penicillamine have higher incidence of adverse effects and neurological deterioration than that of zinc salts. The therapeutic effectiveness of these two regimens does not seem to be significantly different, and these results must be interpreted with caution.

**Systematic Review Registration**: PROSPERO registration, identifier CRD 42021287126.

## 1 Introduction

Wilson disease (WD) is an autosomal recessive inherited disorder caused by pathological copper accumulation in many organs. A wide range of symptoms have been reported, especially hepatic and neurologic symptoms (Bandmann et al., 2015). WD patients may present many different types of liver diseases ranging from transaminase abnormalities, cirrhosis, and even to hepatic failure. Besides, it can also manifest with an impressive spectrum of neurological, behavioral, or psychiatric disorders (Pfeiffer, 2007). The World Health Organization estimates that the global prevalence of WD is 1/10,000–1/30,000 (Liu et al., 2017), but the prevalence may be underestimated. A study from the United Kingdom predicts that the calculated frequency of individuals carrying two mutant pathogenic ATP7B alleles is about 1:7,000, and heterozygote mutations are found in up to 2.5% of the general population (Coffey et al., 2013).

Until now, none of the pharmacological therapies can cure the disease, and WD patients should be treated lifelong. Currently, the drugs used to treat WD are divided into two categories, chelators and zinc salts. Chelators, including d-penicillamine and trientine, reduce copper levels by promoting the urinary excretion of copper, while zinc salts decrease copper uptake from the gastrointestinal tract (Roberts, 2018). Several association guidelines, including the American Association for the Study of Liver Diseases (AASLD) (Roberts et al., 2008), Indian National Association for Study of the Liver (INASL) (Nagral et al., 2019), and the European Association for the Study of the Liver (EASL) (European Association for Study of Liver disease, 2012) guidelines, all recommend d-penicillamine as initial treatment for symptomatic patients with WD, but d-penicillamine is associated with numerous side effects including fever, cutaneous eruptions, neutropenia, thrombocytopenia, and so on (Medici et al., 2006), especially worsening of neurologic symptoms (Seniów et al., 2003). It is generally accepted that zinc salts can be used as initial treatment for presymptomatic WD. Whether the zinc salts can be used to treat symptomatic WD is inconclusive. Zinc has a few side effects, and neurological deterioration seems uncommon in zinc-treated patients (Santiago et al., 2015). In view of the less adverse effects of zinc, some scholars have suggested that WD should be treated with zinc rather than d-penicillamine (Avan et al., 2017). It is doubtful whether this recommendation is reasonable because the difference between d-penicillamine and zinc salts in treatment effectiveness is still inconclusive. Consequently, the comparison between d-penicillamine and zinc salt on both efficacy and safety are urgently needed to guide treatment decision. However, data are limited in individual studies so far. With that in mind, we designed a meta-analysis to clarify the pooled outcomes on the efficacy of d-penicillamine and zinc salt for symptomatic WD patients. In addition, we also assessed the pooled incidence of adverse events and neurological deterioration among the regimens.

## 2 Materials and Methods

Our systematic review and meta-analysis followed a protocol and is reported according to the Preferred Reporting Items for Systematic Reviews and Meta-Analyses (PRISMA) guidelines (Welch et al., 2016; Mikolajewicz and Komarova, 2019). The search strategy, eligibility criteria, and outcomes were registered on the PROSPERO website (CRD 42021287126).

### 2.1 Search Strategy

Two informatics specialists developed the search strategy. We comprehensively and systematically searched the following electronic databases: PubMed, Embase, Cochrane Central Register of Controlled Trials, and Web of Science without language restrictions from inception to October 2021. The used text words (synonyms and word variations) and database-specific subject headings included WD, d-penicillamine, trientine, and Zn. Supplementary Table S1 summarizes the search strategy for PubMed, and it is also applicable for all other databases. We also looked through the reference list from relevant abstracts and original research articles for acquiring other potential studies.

### 2.2 Eligibility Criteria

The current review included cohort studies that enrolled WD patients of any age or stage. Additional eligibility criteria included 1) patients who had received d-penicillamine or zinc salt, and the outcomes of interest were reported, including clinical improvement, adverse events, worsening of neurologic symptoms; 2) both English or non-English publications were eligible.

We excluded the studies if they met one of the following criteria: 1) patients had received combination therapy regimens; 2) patients with fulminant Wilson disease; 3) *in vitro* or animal studies; 4) case report, review, meta-analysis, or only laboratory research; 5) meeting abstracts without available data for analysis. In the situation of two (or more) studies that included the same cohort of patients, the latest one was selected for the current review to avoid the analyses of the duplicated data.

### 2.3 Study Selection and Data Extraction

Two reviewers independently selected articles potentially eligible for inclusion by screening the title and abstract of each article. Included articles were reviewed in full text by each reviewer to produce a list of studies, which were potentially eligible for data extraction. Disagreements were reconciled by the consensus of the corresponding authors.

For each article, two reviewers independently recorded extracted data in a standard form. A third reviewer compared the content and discrepancy on the data extraction. The correspondence authors resolved inconsistencies by reviewing the full text of the articles. We extracted the following data: the first author’s name, year of publication, study country, patient population, dosage, and duration of treatment drugs. Outcome data included “improved” and adverse events including dermatologic reactions, nephropathies, hematologic toxicity, and worsening of neurological symptoms. Because of a high heterogeneity in outcome reporting, we did not stipulate a specific “improved” standard but, instead, used judgment on whether the interventional drug improves neurological or liver-related symptoms in the included article. When study cohorts included patients who experienced drug switch, we considered patient data only for the first treatments. If the outcome was not reported at the time of drug switch, we censored the patient from that outcome analysis. If the missing data in the article is not indispensable, we ignored it.

### 2.4 Assessment of the Study Quality and Risk of Bias

The quality of each study was independently assessed by two reviewers. The risk of bias for cohort studies and respective studies was assessed using the modified Newcastle–Ottawa scale (NOS). With NOS tools, observational studies were scored as follows: selection (up to four points), comparability (up to two points), and exposure or outcome of study participants (up to three points). Studies with a cumulative score of ≥7, 4–6, and <4 were considered as high, fair, and low quality.

### 2.5 Statistical Analysis

We performed a meta-analysis using the STATA 12.0 software. All data were expressed as the combination of relative risk (RR) and 95% CI, and *p* < 0.05 was considered statistically significant. To measure the overall heterogeneity across the included studies, we used the Cochrane Q test and *I* ^2^ statistic, where *I* ^2^ value >50% or the *p*-value of Cochrane Q test <0.1 suggests a significant heterogeneity. The random-effect model was adopted for the high heterogeneity; otherwise, a fixed-effect model was employed. To explore the effect of a single study on overall results, sensitivity analysis was performed by removing one study sequentially to evaluate its effect on the overall results including all published articles selected for our study. The Egger’s regression asymmetry test and funnel plot were employed to assess publication bias, with *p*  < 0.05 considered to present statistical significance.

## 3 Results

### 3.1 Results of the Search

**FIGURE 1:**
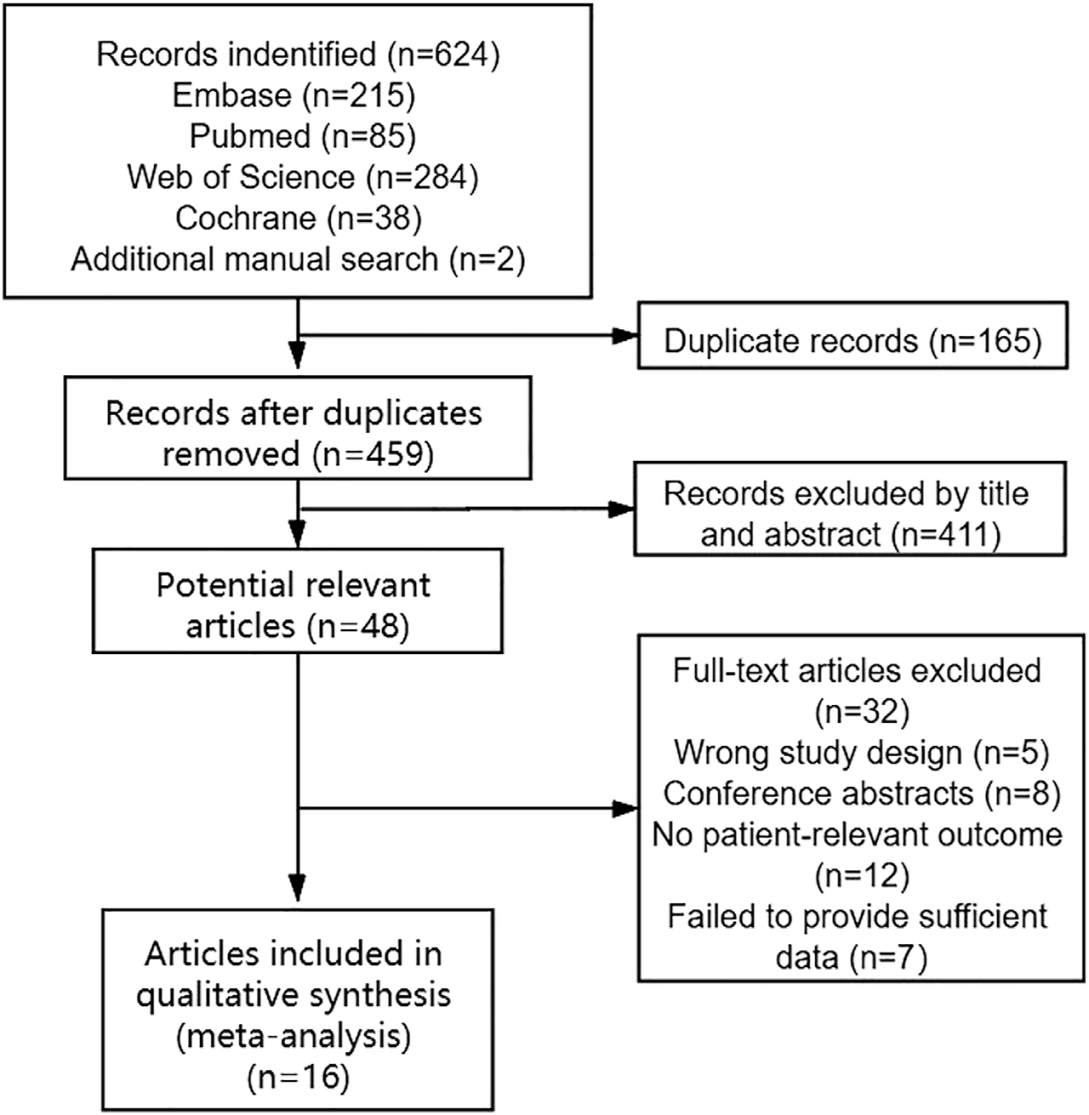
Flow diagram for the selection of studies.

In total, 622 studies were identified through the initial search in the electronic databases. Moreover, two potentially eligible records were found by manual research, of which, 165 studies were excluded as duplicates. After further title and abstract review, 411 reports were excluded because they were irrelevant to this meta-analysis. After that, we continued to screen the remaining 48 potentially eligible studies by full text. Sixteen studies met the inclusion criteria and were selected for further analysis. Figure 1 shows our selection process and reasons for the exclusions.

### 3.2 Characteristics of the Studies

The included studies were published between 1996 and 2021. The characteristics of the included studies are summarized in Table 1. Among 16 cohort studies, 13 were considered high quality with a cumulative NOS score of seven points or greater, whereas the remaining three studies had medium quality with score of 4–6 points. The risk of bias comprehensive assessment is shown in Supplementary Table S2.

**TABLE 1:** The characteristics of the included studies.

Author	Year	Country	Study design	Patient population	Treatment	Outcome	Patients (*n*) (original size)
Couchonnal (Couchonnal et al., 2021)	2021	France	Retrospective and prospectively study	WD diagnosis 1995–2019	1. d-Penicillamine	Side effects (1)	17 [131]
				Age: 10.7 ± 4.2 (1–18)	2. Zinc salts	Side effects (2)	0 [24]
				Presentation: neurologic; hepatic symptoms; asymptomatic	3. Trientine	Side effects (3)	0 [17]
						Improved (1)	109 [131]
						Improved (2)	10 [18]
Zhang (Zhang et al., 2020)	2020	China	Retrospective study	WD diagnosis 2015–2018	1. d-Penicillamine(5 mg/kg/d)	Neurological improved (1)	37 [65]
				Age: 24.47 ± 8.00			
				Presentation: neurologic			
Zhou (Zhou et al., 2020)	2020	China	Prospectively study	WD diagnosis 2015–2019	1. d-Penicillamine	Side effects (1)	13 [38]
				Presentation: neurologic; hepatic symptoms	2. Zinc gluconate	Side effects (2) Neurological improved (1)	5 [10]
							13 [38]
Mayr (Mayr et al., 2021)	2020	Germany	Retrospective study	WD diagnosis 1980–1992	1. d-Penicillamine	Side effects (1)	24 [26]
				Age: <18	2. Trientine	Side effects (2)	10 [27]
				Presentation: neurologic; hepatic symptoms			
Litwin (Litwin et al., 2015)	2015	Poland	Retrospective study	WD diagnosis 2005–2009	1. d-Penicillamine	Neurological deterioration (1)	12 [42]
				Presentation: neurologic	2. Zinc sulfate	Neurological deterioration (2)	4 [28]
Kalita (Kalita et al., 2015)	2015	India	Cohort study	Age: 11 (5–37)	1. d-Penicillamine (750 mg/day)	Neurological deterioration (1)	12 [42]
				Presentation: neurologic	2. Elemental zinc (150 mg/day)	Neurological deterioration (2)	0 [9]
Członkowska (Członkowska et al., 2014)	2014	Poland	Retrospective study	WD diagnosis 2005–2009	1. d-Penicillamine	Neurological deterioration (1)	4 [35]
				Presentation: neurologic; hepatic symptoms	2. Zinc sulfate	Neurological deterioration (2)	1 [21]
						Neurological improved (1)	29 [35]
						Neurological improved (2)	15 [21]
						Hepatic improved (1)	34 [36]
						Hepatic improved (2)	48 [51]
Sini (Sini et al., 2013)	2013	Italy	Cohort study	WD diagnosis 1970–2000	1. d-Penicillamine (600–1,200 mg/day)	Hepatic improved (1)	12 [13]
				Presentation: hepatic symptoms	2. Elemental zinc	Hepatic improved (2)	3 [3]
					(150–300 mg/day)		
Rodriguez (Rodríguez et al., 2012)	2012	Spain	Retrospective study	Age: 22 (6–50)	1. d-Penicillamine	Side effects (1)	4 [18]
				Presentation: neurologic; hepatic symptoms	2. Zinc salts	Side effects (2)	0 [2]
						improved (1)	11 [18]
						improved (2)	2 [2]
Weiss (Weiss et al., 2011)	2011	Germany	Retrospective cohort study	Presentation: neurologic; hepatic symptoms; asymptomatic	1. d-Penicillamine	Side effects (1)	99 [313]
					2. Zinc salts	Side effects (2)	10 [88]
					3. Trientine	Side effects (3)	9 [102]
						Neurological deterioration (1)	22 [243]
						Neurological deterioration (2)	9 [95]
Masebas (Maselbas et al., 2010)	2010	Poland	Retrospective study	Presentation: neurologic; hepatic symptoms; asymptomatic	1. d-Penicillamine	Neurological deterioration (1)	6 [55]
					2. Zinc sulfate	Neurological deterioration (2)	3 [47]
						improved (1)	44 [55]
						improved (2)	37 [47]
Yokoyama (Cope-Yokoyama et al., 2010)	2010	Italy	Cohort study	WD diagnosis 1981–2006	1. d-Penicillamine	Hepatic improved (1)	3 [5]
				Age: 17.3 (6–35)	2. Zinc sulfate	Hepatic improved (2)	3 [7]
				Presentation: hepatic symptoms			
Bruha (Bruha et al., 2011)	2010	Czech Republic	Retrospective study	WD diagnosis 1965–2008	1. d-Penicillamine (600–1,200 mg/day)	Neurological deterioration (1)	2 [50]
				Age: 38.5 ± 11 (16–63)	2. Elemental zinc	Neurological deterioration (2)	0 [3]
				Presentation: neurologic; hepatic symptoms; asymptomatic	(150 mg/day)	Neurological improved (1)	36 [50]
						Neurological improved (2)	2 [3]
						Hepatic improved (1)	32 [40]
						Hepatic improved (2)	7 [8]
Merle (Merle et al., 2007)	2007	Germany	Cohort study	WD diagnosis 2000–2005	1. d-Penicillamine (900–1,800 mg/day)	Side effects (1)	78 [138]
				Age: 20.4 ± 10.6 (4–56)	2. Trientine	Side effects (2)	4 [140]
				Presentation: neurologic; hepatic symptoms; asymptomatic	(900–2,100 mg/day)	Side effects (3)	19 [140]
					3. Elemental zinc	Neurological deterioration (1)	19 [138]
					(150–250 mg/day)	Neurological deterioration (3)	6 [140]
Medici (Medici et al., 2006)	2006	Italy	Retrospective study	WD diagnosis 1980–2005	1. d-Penicillamine (600–1,800 mg/day)	Neurological deterioration (1)	6 [8]
				Age: 15.5 ± 7.2 (4–35)	2. Zinc sulfate	Neurological deterioration (2)	1 [10]
				Presentation: neurologic; hepatic symptoms	(660–880 mg/day)	Neurological improved (1)	2 [8]
						Neurological improved (2)	9 [10]
						Hepatic improved (1)	7 [17]
						Hepatic improved (2)	10 [20]
Czlonkowska (Czlonkowska et al., 1996)	1996	Poland	Retrospective study	WD diagnosis 1980–1992	1. d-Penicillamine (1,000–1,500 mg/day)	Side effects (1)	15 [34]
				Presentation: neurologic; psychiatric; hepatic symptoms; asymptomatic	2. Zinc sulfate	Side effects (2)	4 [33]
					(600–800 mg/day)	Neurological deterioration (1)	3 [34]
						Neurological deterioration (2)	2 [33]
						improved (1)	13 [19]
						improved (2)	23 [29]

### 3.3 Treatment Effectiveness for Wilson Disease Patients

#### 3.3.1 Overall Treatment Effectiveness Results

**FIGURE 2:**
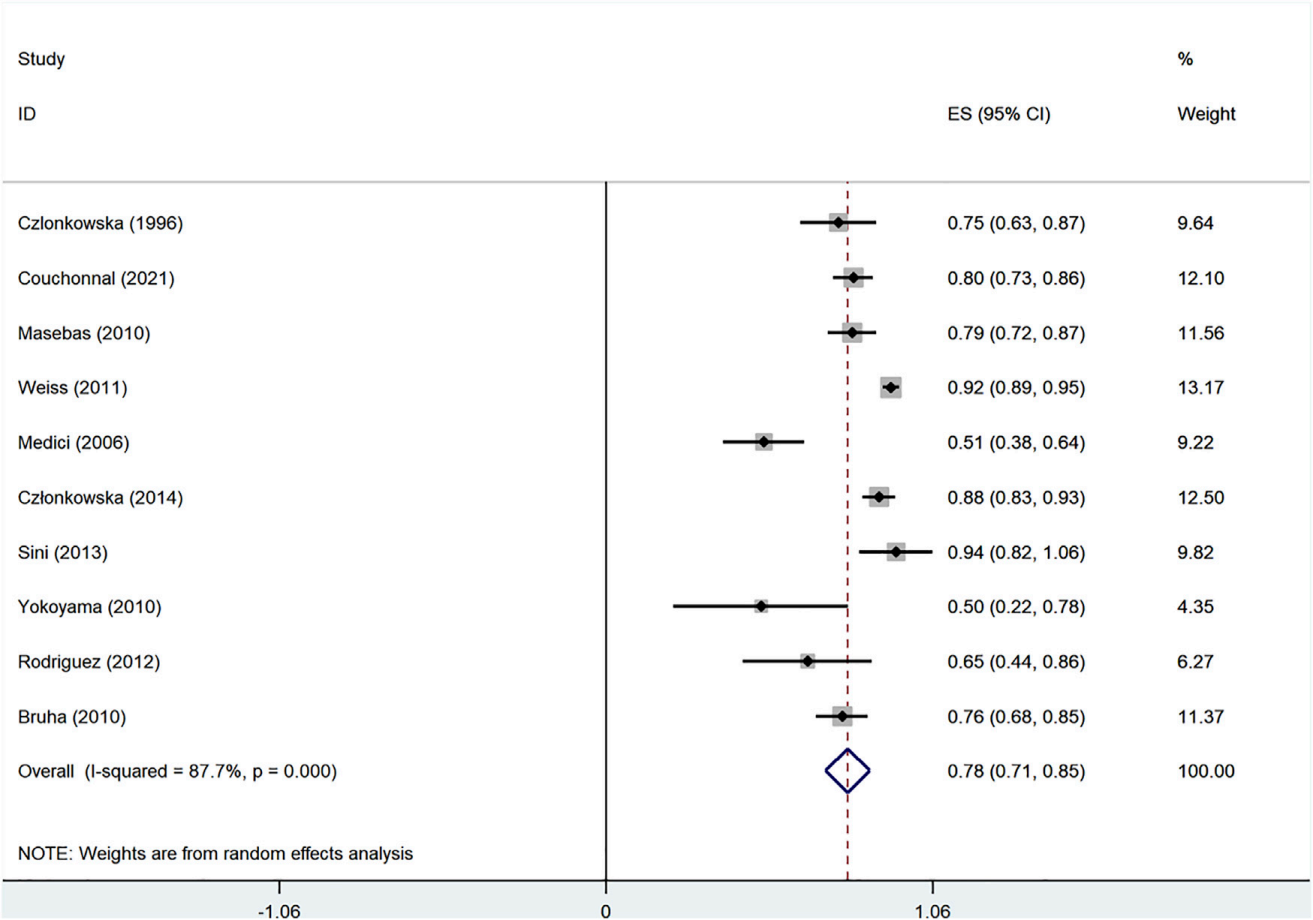
Forest plot of the pooled improved rate for all studied Wilson disease (WD) patients.

As shown in Figure 2, the pooled improved rate for all 1,047 WD patients in the 11 studies (Czlonkowska et al., 1996; Medici et al., 2006; Cope-Yokoyama et al., 2010; Maselbas et al., 2010; Bruha et al., 2011; Weiss et al., 2011; Rodríguez et al., 2012; Sini et al., 2013; Członkowska et al., 2014; Couchonnal et al., 2021) was 78.0% (95% CI: 70.8%–85.2%). The random-effect model was adopted in the analyses because the *I* ^2^ was 87.7% (*p* < 0.01). For comparison of improved rate in WD patients treated with d-penicillamine and zinc salts, we employed the fixed-effect model to compile the improved data from 11 studies including 740 patients treated with d-penicillamine and 307 patients treated with zinc salts. The result showed that no significant difference was found between the two groups (RR: 1.07, 95% CI: 0.99%–1.15%; *p* = 0.069, Supplementary Figure S1).

#### 3.3.2 Treatment Effectiveness Results For Symptomatic Hepatic Wilson Disease Patients

As shown in Supplementary Figure S2, the pooled improved rate for all symptomatic hepatic WD patients was 76.0% (95% CI: 59.0%–92.0%). In order to compare the treatment effectiveness of d-penicillamine and zinc salts for patients with hepatic WD, five studies (Medici et al., 2006; Cope-Yokoyama et al., 2010; Bruha et al., 2011; Sini et al., 2013; Członkowska et al., 2014), which enrolled 111 patients treated with d-penicillamine and 89 patients treated with zinc salts, were analyzed. The fixed-effect model was adopted in the analyses because the *I* ^2^ was 0.00% (*p* = 0.90). The difference between the two groups was not statistically significant (RR: 0.98, 95% CI: 0.86%–1.12%; *p* = 0.765, Supplementary Figure S3).

#### 3.3.3 Treatment Effectiveness Results For Neurological Wilson Disease Patients

**FIGURE 3:**
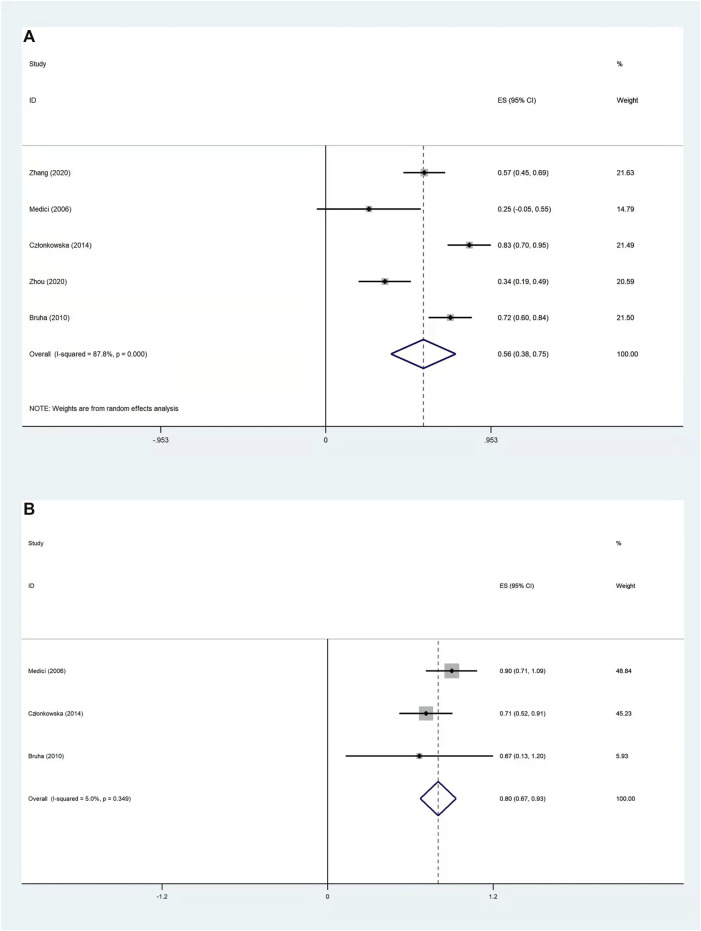
Forest plot of the pooled improved rate for neurological WD patients treated with d-penicillamine **(A)** and zinc salts **(B)**.

To compare the effectiveness of d-penicillamine and zinc salts for neurological WD patients, we performed a meta-analysis concerning RRs in terms of treatment regimens. Three studies (Członkowska et al., 2014; Bruha et al., 2011; Medici et al., 2006) containing 93 patients treated with d-penicillamine and 34 patients treated with zinc salts were analyzed. The results showed that the pooled improved rate for all neurological WD patients was 74.0% (95% CI: 66.0%–81.0%, Supplementary Figure S4) and there was no significant difference between the two groups (RR: 0.83%, 95% CI: 0.40%–1.75%; *p* = 0.632, Supplementary Figure S5). We further analyzed five studies (Medici et al., 2006; Bruha et al., 2011; Członkowska et al., 2014; Zhang et al., 2020; Zhou et al., 2020) that reported effectiveness data in terms of neurological WD patients (*n* = 196) treated with d-penicillamine, and the pooled improved rate was 56.3% (95% CI: 37.5%–75.1%, Figure 3). In addition, we calculated the pooled improved rate in three studies that reported the effectiveness of zinc salt treatment for neurological WD patients (*n* = 34). The pooled improved rate was 80.2% (95% CI: 67.2%–93.2%, Figure 3).

### 3.4 Treatment Safety for Wilson Disease Patients

#### 3.4.1 Treatment Adverse Effects

**FIGURE 4:**
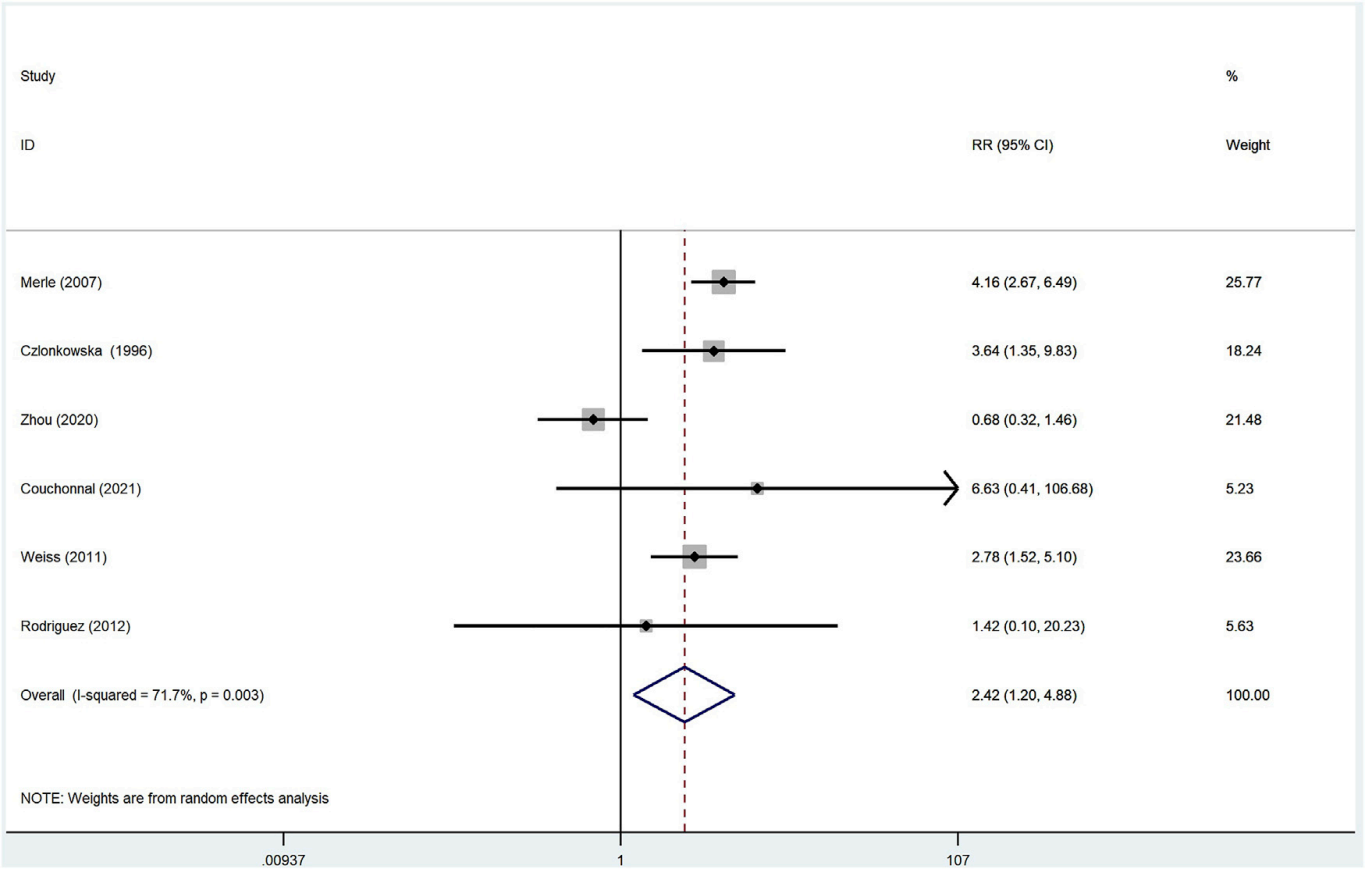
Meta-analysis of adverse effects in WD patients treated with d-penicillamine compared with zinc salts.

Of the 16 studies reviewed, there were six studies (Czlonkowska et al., 1996; Merle et al., 2007; Weiss et al., 2011; Rodríguez et al., 2012; Zhou et al., 2020; Couchonnal et al., 2021) that reported adverse effect data in a total of 672 patients treated with d-penicillamine and 297 patients treated with zinc salts. When assessing the overall heterogeneity across the study on the side effect data collection, the *I* ^2^ was >50%, which indicated high heterogeneity. Thus, the random-effect model was adopted for the analysis of side effects across the studies. In WD patients treated with d-penicillamine, the incidence of adverse events was higher than those treated with zinc salts (RR: 2.42, 95% CI: 1.20%–4.88%; *p* = 0.014, Figure 4).

#### 3.4.2 Neurological Deterioration After Treatment

**FIGURE 5:**
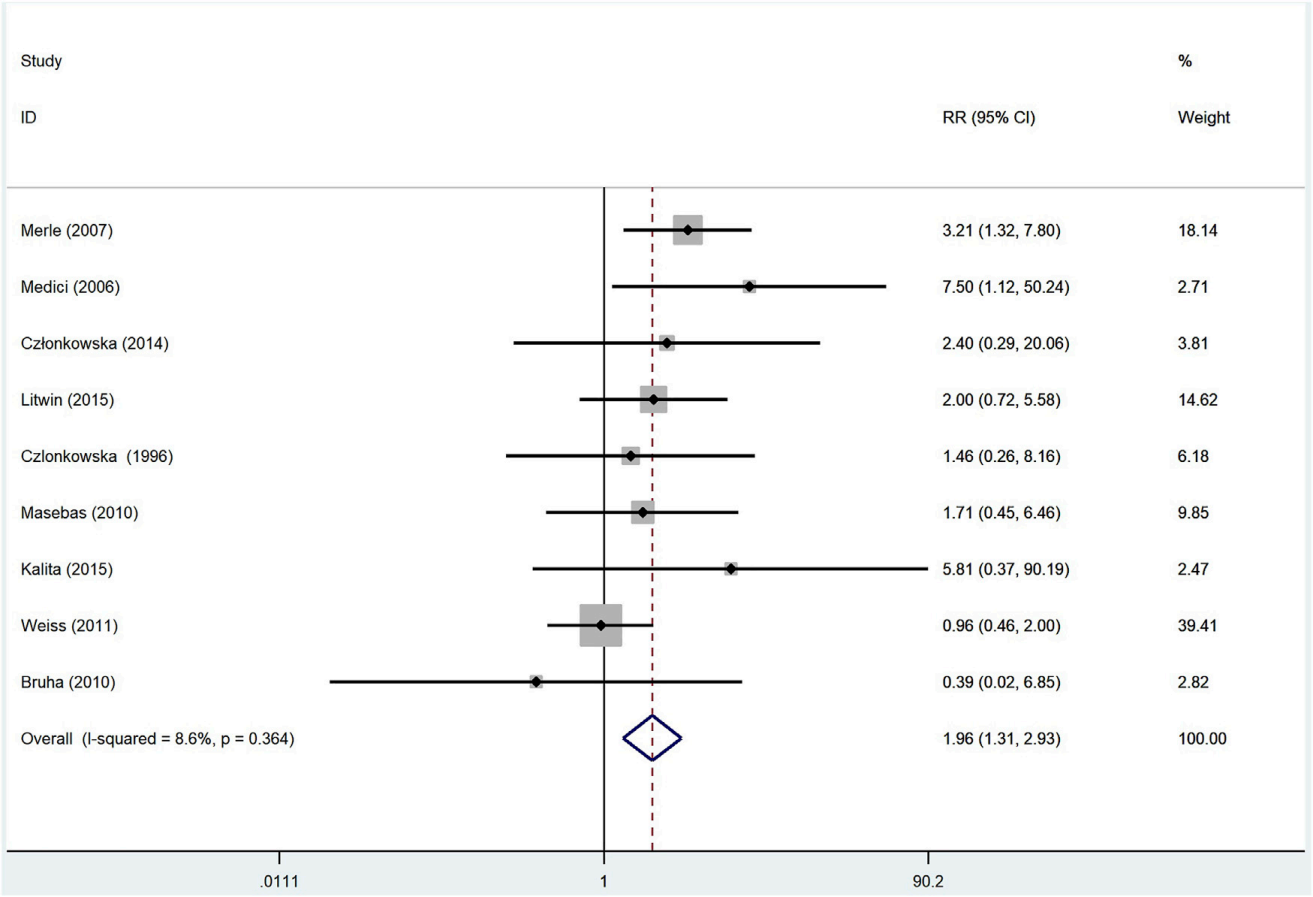
Meta-analysis of neurological deterioration in WD patients treated with d-penicillamine compared with zinc salts.

To gain a better understanding of the neurological deterioration in WD patients, we compared the pooled neurological deterioration data of patients treated with d-penicillamine and zinc salts. The meta analysis included nine studies (Czlonkowska et al., 1996; Medici et al., 2006; Merle et al., 2007; Maselbas et al., 2010; Bruha et al., 2011; Weiss et al., 2011; Członkowska et al., 2014; Litwin et al., 2015; Kalita et al., 2015), and fixed-effect model was adopted because the *I* ^2^ was 8.6%. As shown in Figure 5, patients who were treated with d-penicillamine (*n* = 647) had a significantly higher frequency of neurological deterioration than those treated with zinc salts (*n* = 386) (RR: 1.96, 95% CI: 1.31%–2.93%; *p* = 0.001).

### 3.5 Sensitivity Analysis

To estimate the influence of single study on overall results of meta-analysis, we conducted sensitivity analysis as presented in Supplementary Figure S6. The analysis showed that the pooled results were not significantly changed after deleting each trial, which confirmed the rationality and reliability of our meta-analysis.

### 3.6 Publication Bias

Egger’s plot (Supplementary Figure S7) showed that the current meta-analysis had no significant publication bias (*p* = 0.23), and the funnel plot of the included studies was symmetrical (Supplementary Figure S8), which provides further evidence for no publication bias.

## 4 Discussion

d-penicillamine and zinc salts are recommended by INASL (Nagral et al., 2019), AASLD (Roberts et al., 2008), and EASL (European Association for Study of Liver disease, 2012) guidelines for the treatment of patients with WD. Our meta-analysis revealed that the pooled improved rate of the regimens in all symptomatic WD patients is 78.0% (95% CI: 70.8%–85.2%), and in symptomatic hepatic WD and neurological WD patients, the pooled improved rates are 76.0% (95% CI: 59.0%–92.0%) and 74.0% (95% CI: 66.0%–81.0%), respectively.

The use of zinc in the treatment of patients with symptomatic WD is still controversial (Członkowska et al., 2005; Kasztelan-Szczerbinska and Cichoz-Lach, 2021). In the present meta-analysis, we aimed to assess and compare the effectiveness and safety of d-penicillamine and zinc salt therapies for symptomatic WD patients. In the effectiveness analysis, our results showed that WD patients treated with d-penicillamine and zinc seem to exhibit similar treatment effectiveness in all symptomatic WD patients (RR: 1.07, 95% CI: 0.99%–1.15%; *p* = 0.069). These two treatment options have similar effects in treating symptomatic hepatic WD either (RR: 0.98, 95% CI: 0.86%–1.12%; *p* = 0.765). Our results are consist with a study in a Polish cohort (Lucena-Valera et al., 2021), which showed that there are no differences in survival of WD patients who started therapy with zinc sulfate or d-penicillamine. Nevertheless, this finding was questioned by other researchers. For example, Weiss et al. (2011) conducted a retrospective analysis of 288 WD patients in Europe, and the results showed that zinc monotherapy is not as effective as chelators in preventing hepatic deterioration. Our meta-analysis provides evidence for the effectiveness of zinc salts in the treatment of symptomatic hepatic WD. According to the results of the studies we included, most patients were treated with zinc sulfate, and the dosages equivalent to zinc element is 150–250 mg/day. This is consistent with the EASL guideline recommendation.

We also compared the treatment effectiveness of d-penicillamine and zinc salts for neurological WD patients, and the results showed that there is no difference in improved rate between the two groups (RR: 0.83, 95% CI: 0.40%–1.75%; *p* = 0.632). Nevertheless, the pooled improved rate in five studies that reported effectiveness data in terms of neurological WD patients who were treated with d-penicillamine was 56.3% (95% CI: 37.5%–75.1%). The pooled improved rate in three studies that reported zinc salt treatment for neurological WD patients was 80.2% (95% CI: 67.2%–93.2%). As a result, zinc salts seem to be superior to d-penicillamine in improving effectiveness for neurological WD patients. Our results are in accordance with the study by Linn et al. (2009), which showed that the outcome of exclusive zinc therapy is generally satisfied in cases with neurologic disease. This also confirmed the recommendation in the EASL guidelines, which put forward that zinc may be used as a first-line therapy for neurological patients.

In addition, we compared the adverse effects of d-penicillamine and zinc salts. The results showed that the adverse effect incidence of d-penicillamine is higher than that of zinc salts (RR: 2.42, 95% CI: 1.20%–4.88%; *p* = 0.014), which is consistent with our previous cognition. In terms of the neurological deterioration, our analysis result documented that the incidence is higher in patients treated with d-penicillamine than those treated with zinc salts (RR: 1.96, 95% CI: 1.31 –2.93 ; *p* = 0.001). However, Litwin et al. (2015) found that neurological worsening at the beginning of anti-copper therapy does not differ between d-penicillamine and zinc groups. Of note, this article belongs to a single-center retrospective study. Our result aggregated multiple data, so it seems to be more convincing.

We also analyzed the occurrence of adverse effects in WD patients treated with d-penicillamine and trientine. There were four studies (Couchonnal et al., 2021; Mayr et al., 2021; Weiss et al., 2011; Merle et al., 2007), including 608 patients treated with d-penicillamine and 286 patients treated with trientine, that compared the occurrence of adverse effects between the two treatment regimens. The result showed that the adverse effects of trientine is lower than that of d-penicillamine (RR: 5.19, 95% CI: 1.79%–15.05%; *p* = 0.002, Supplementary Figure S9), which is consistent with EASL guideline recommendations. Since only one study (Couchonnal et al., 2021) in the included literatures compared the treatment effectiveness of d-penicillamine and trientine, we did not conduct a meta-analysis. The data in this study show that the improved rate of d-penicillamine and trientine is 109/131 and 6/17, respectively. This is not powerful enough to indicate the difference in treatment effectiveness between the two regimens. Therefore, more cohort studies are needed in the future.

To the best of our knowledge, this is the first meta-analysis that compared the efficacy and safety of chelators and zinc salt treatment in both symptomatic hepatic and neurological WD patients. In a previous meta-analysis performed by Appenzeller-Herzog et al. (2019), they did not perform meta-analysis in different categories: symptomatic hepatic WD and neurological WD, which lead to the loss of the opportunity to better assess the efficacy of treatment.

Several limitations are noted in our study. First, there are no randomized controlled trails (RCTs) in the published studies, which results in unbalanced study arms. The number of patients treated with d-penicillamine in the included studies is greater than that of patients treated with zinc salts, which limits the ability to derive definitive conclusions regarding the safety and efficacy of a regimen. In the future, we still need to design RCTs to increase the comparability among groups, therefore, reducing selection bias. Second, individual meta-analysis merged multiple studies, which bring about the heterogeneity. Unfortunately, due to the limitation of the number of included studies, we were not able to perform subgroup analysis. As a result, we adopted random-effect model to merge these heterogeneous studies. Third, the severity of the adverse effects is heterogeneous. For example, rash can be improved by stopping the drug, while the nephrotoxicity is more serious and irreversible. However, due to the lack of specific data, we are uncapable to stratify data according to the different degrees of adverse effects. Fourth, in some centers, both d-penicillamine and zinc salts were used as initial therapy. For example, in the study by Czlonkowska et al. (Kasztelan-Szczerbinska and Cichoz-Lach, 2021), zinc or d-penicillamine can be used as initial therapy in symptomatic WD patients regardless of disease severity, but in some centers, zinc was not used for the first-line treatment of symptomatic WD patients. For example, the study of Weiss et al. (2011) pointed out that zinc therapy was only used as second- or third-line therapy. First-line therapy with zinc was used in patients with a neurologic presentation. These may affect the balance of patients. All in all, these results mentioned above must be interpreted with caution.

Despite the above limitations, the strength of our meta-analysis lies in its exhaustive literature research, well-defined approaches for data selection/extraction, comprehensive statistical analyses, and reporting in accordance with PRISMA statements with no significant evidence of publication bias.

In conclusion, our meta-analysis showed that pharmacological therapies have a relatively high pooled improved rate for WD patients. In addition, patients treated with d-penicillamine have higher incidence of adverse effects and neurological deterioration than those treated with zinc salts, and the therapeutic effectiveness of these two regimens does not seem to be significantly different. Our findings provide further support for the recommendation in the EASL guidelines and offer reliable evidence for clinical decision.
