# Supplementary material for: Comparison of the Effectiveness and Safety of d-Penicillamine and Zinc Salt Treatment for Symptomatic Wilson Disease: A Systematic Review and Meta‐Analysis
Source: Front Pharmacol. 2022 Mar 18;13:847436. doi: 10.3389/fphar.2022.847436 (PMC8975209; doi:10.3389/fphar.2022.847436)
Supplement: Supplementary file 1 [file DataSheet1.docx]

**Supplementary table**

**Table S1 PubMed search strategy**

| **Number** | **Search items** |
| --- | --- |
| #1 | "Hepatolenticular Degeneration"[Mesh] OR Hepatolenticular Degeneration*[tw] OR Pseudosclerosis[tw] OR "Wilson Disease*"[tw] OR "Wilson's Disease*"[tw] OR "Wilsons Disease*"[tw] OR "Westphal-Strumpell Syndrome*"[tw] OR "Copper Storage Disease*"[tw] OR "Progressive Lenticular Degeneration*"[tw] OR Neurohepatic Degeneration*[tw] |
| #2 | "Zinc Compounds"[Mesh] OR Zincteral[tw] OR Zinc[tw] |
| #3 | "Penicillamine"[Mesh] OR Penicillamin*[tw] OR Mercaptovaline[tw] OR Dimethylcysteine[tw] OR Metalcaptase[tw] OR Cuprimine[tw] OR Cuprenil[tw] OR Acetylpenicillamine[tw] OR "S-NONAP"[tw] OR atamir[tw] OR pendramine[tw] |
| #4 | #4. "Trientine"[Mesh] OR Trientine[tw] OR Triethylenetetramine[tw] OR Trien[tw] OR Syprine[tw] OR teta[tw] |
| #5 | #1 AND (#2 OR #3 OR #4) |
| #6 | ("controlled clinical trial"[pt] OR "Controlled Clinical Trials as Topic"[MeSH] OR "Random Allocation"[MeSH] OR "Double-Blind Method"[MeSH] OR "single-blind method"[MeSH] OR "Control Groups"[MeSH] OR "cross-over studies"[MeSH] OR random*[tiab] OR placebo[tiab] OR trial[tiab] OR groups[tiab] OR crossover[tiab] OR cross-over[tiab]) NOT ("Animals"[Mesh] NOT ("Humans"[Mesh] AND "Animals"[Mesh])) |
| #7 | #5AND #6 |

**Table S2 Evaluation of the quality of cohort studies included in the meta analysis (assessed by Newcastle-Ottawa scale)**

| Author | Year | Study population selection | | | | Comparability between groups | Outcome measurement | | Total score (9) |
| --- | --- | --- | --- | --- | --- | --- | --- | --- | --- |
|  |  | Representativeness of the sample  （1） | Determination of treatment  （1） | Make sure that the research started without  Outcome indicators to be observed  （1） | Identify patients in the non-exposed group  （1） | Subjects are comparable in design and statistical analysis  （2） | Result evaluation  （2） | Adequacy of follow-up time after the results occurred  （1） |  |
| Merle | 2007 | 1 | 1 | 1 | 0 | 2 | 2 | 1 | 8 |
| Zhang | 2020 | 1 | 1 | 0 | 0 | 2 | 2 | 1 | 7 |
| Medici | 2006 | 1 | 1 | 1 | 0 | 2 | 2 | 1 | 8 |
| Członkowska | 2014 | 1 | 1 | 1 | 0 | 2 | 2 | 1 | 8 |
| Litwin | 2015 | 0 | 1 | 0 | 0 | 2 | 2 | 1 | 6 |
| Czlonkowska | 1996 | 1 | 1 | 1 | 0 | 1 | 2 | 1 | 7 |
| Sini | 2013 | 1 | 1 | 1 | 0 | 2 | 2 | 1 | 8 |
| Zhou | 2020 | 1 | 1 | 1 | 1 | 1 | 2 | 0 | 7 |
| Mayr | 2020 | 1 | 0 | 1 | 0 | 1 | 2 | 1 | 6 |
| Couchonnal | 2021 | 1 | 1 | 1 | 0 | 2 | 2 | 1 | 8 |
| Masebas | 2010 | 1 | 0 | 0 | 0 | 2 | 2 | 1 | 6 |
| Kalita | 2015 | 1 | 1 | 0 | 0 | 2 | 2 | 1 | 7 |
| Yokoyama | 2010 | 1 | 1 | 1 | 0 | 2 | 2 | 1 | 8 |
| Bruha | 2010 | 1 | 1 | 1 | 0 | 1 | 2 | 1 | 7 |
| Weiss | 2011 | 1 | 1 | 1 | 0 | 1 | 2 | 1 | 7 |
| Rodriguez | 2012 | 1 | 1 | 1 | 0 | 1 | 2 | 1 | 7 |

**Supplementary figure**

**
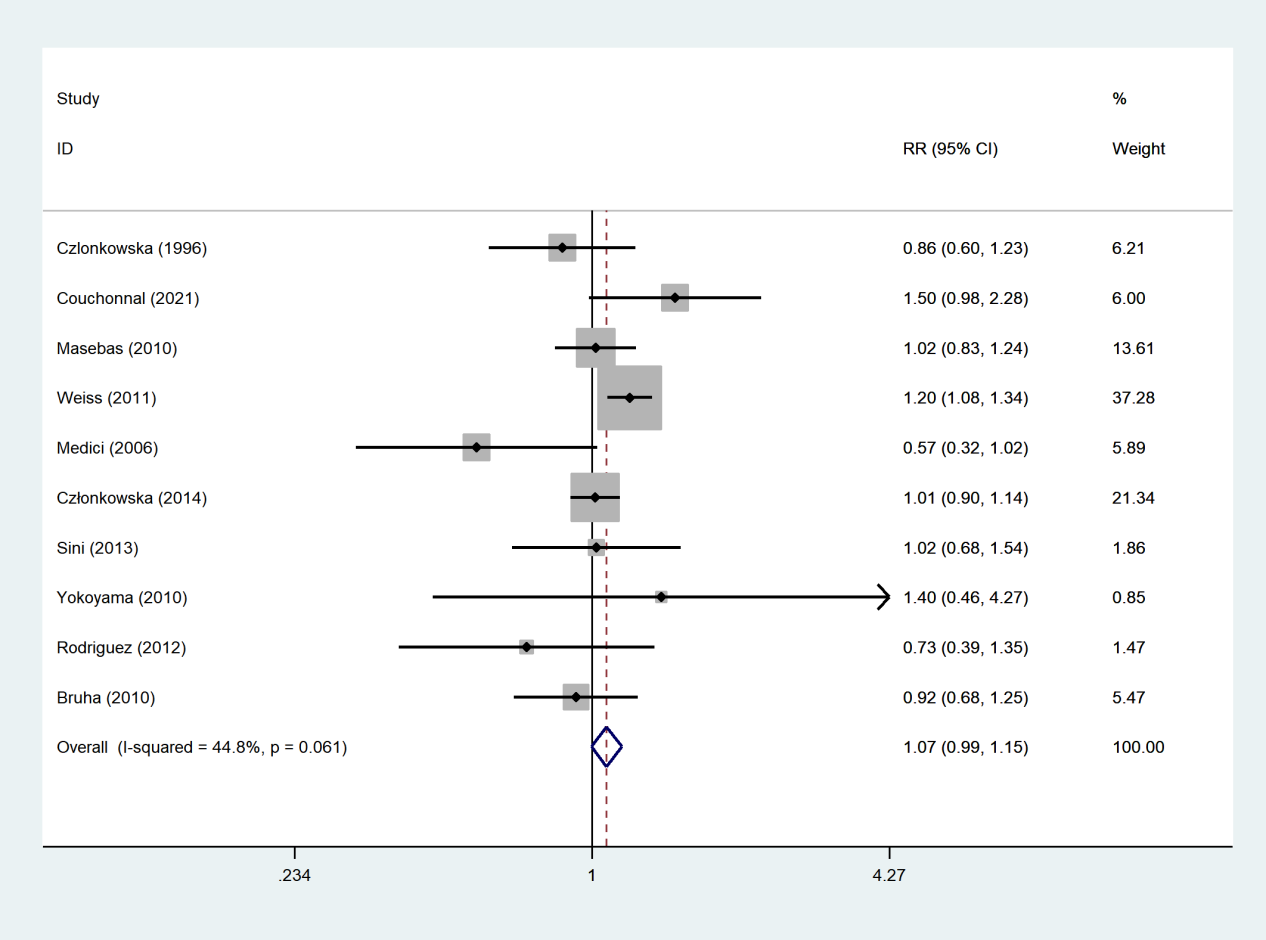
**

**Figure S1. Forest plot of the WD patients treated with penicillamine compared with zinc salts.**

**
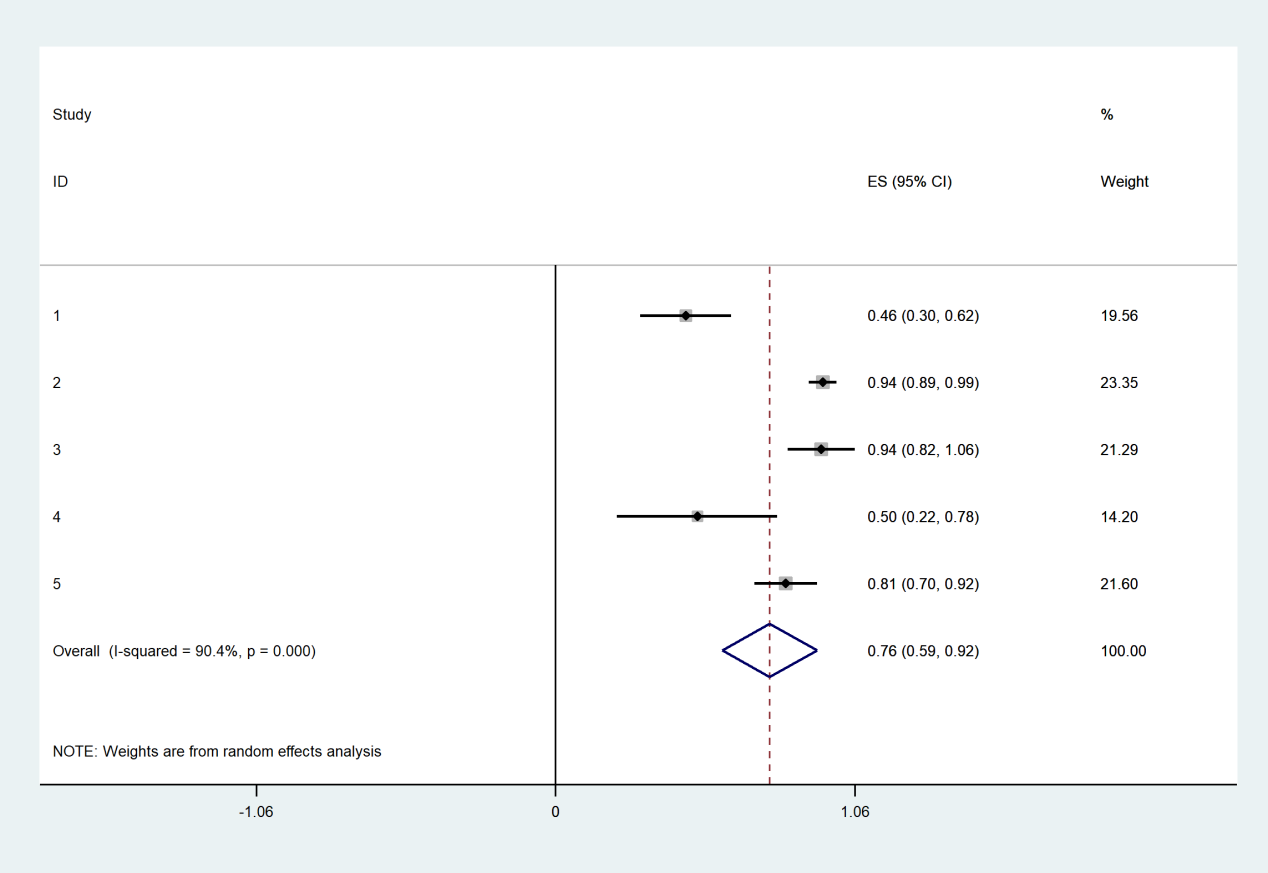
**

**Figure S2. Forest plot of the pooled improved rate for hepatic WD patients.**

**
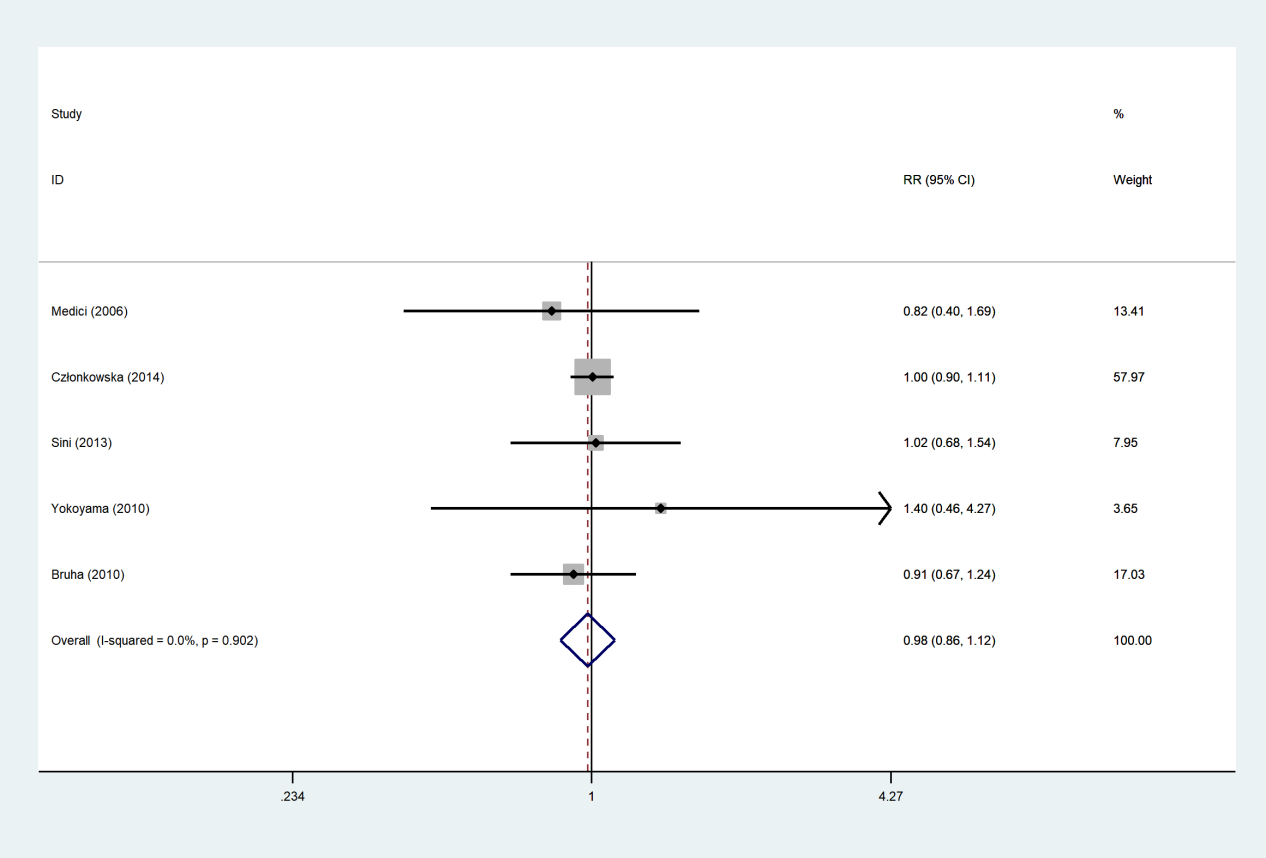
**

**Figure S3. Meta-analysis of treatment effectiveness in hepatic WD patients treated with penicillamine compared with zinc salts.**

**
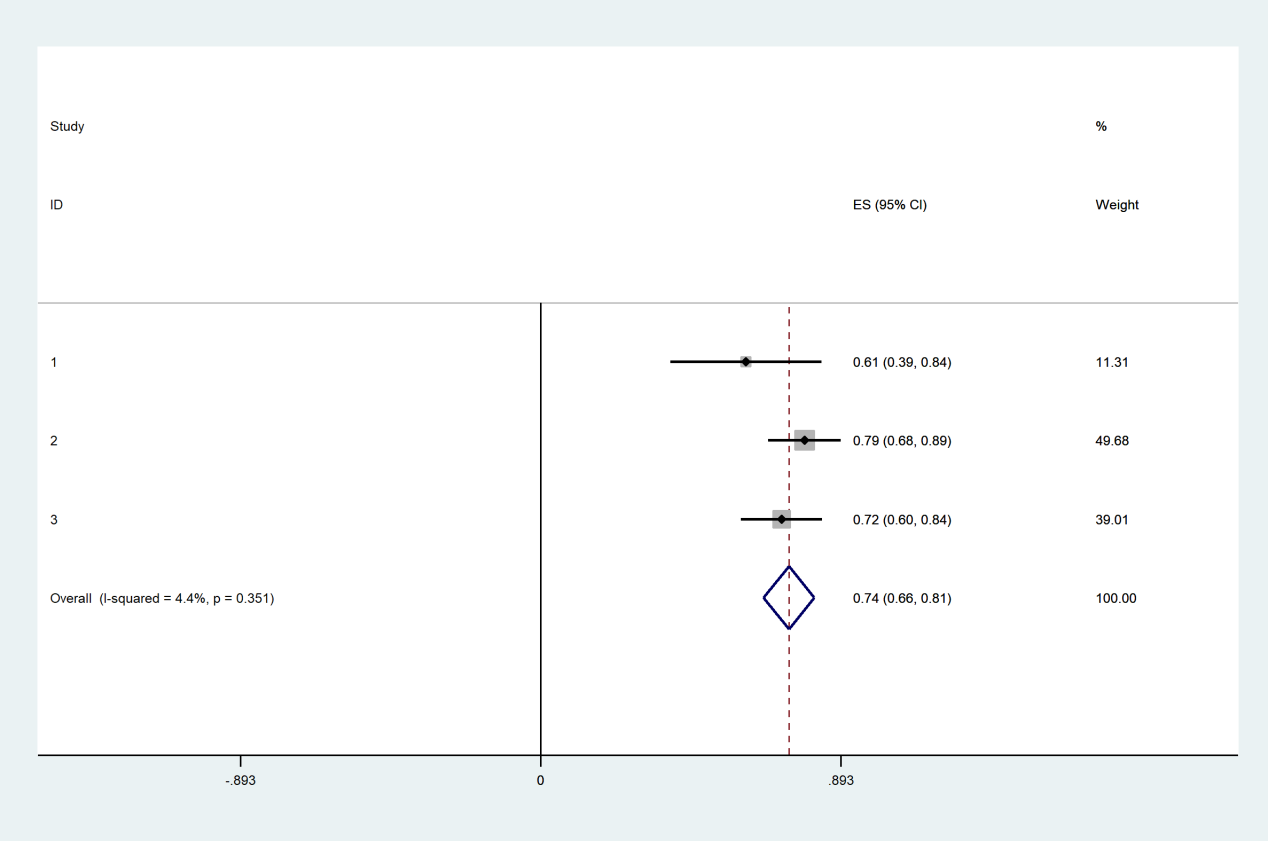
**

**Figure S4. Forest plot of the pooled improved rate for neurological WD patients.**

**
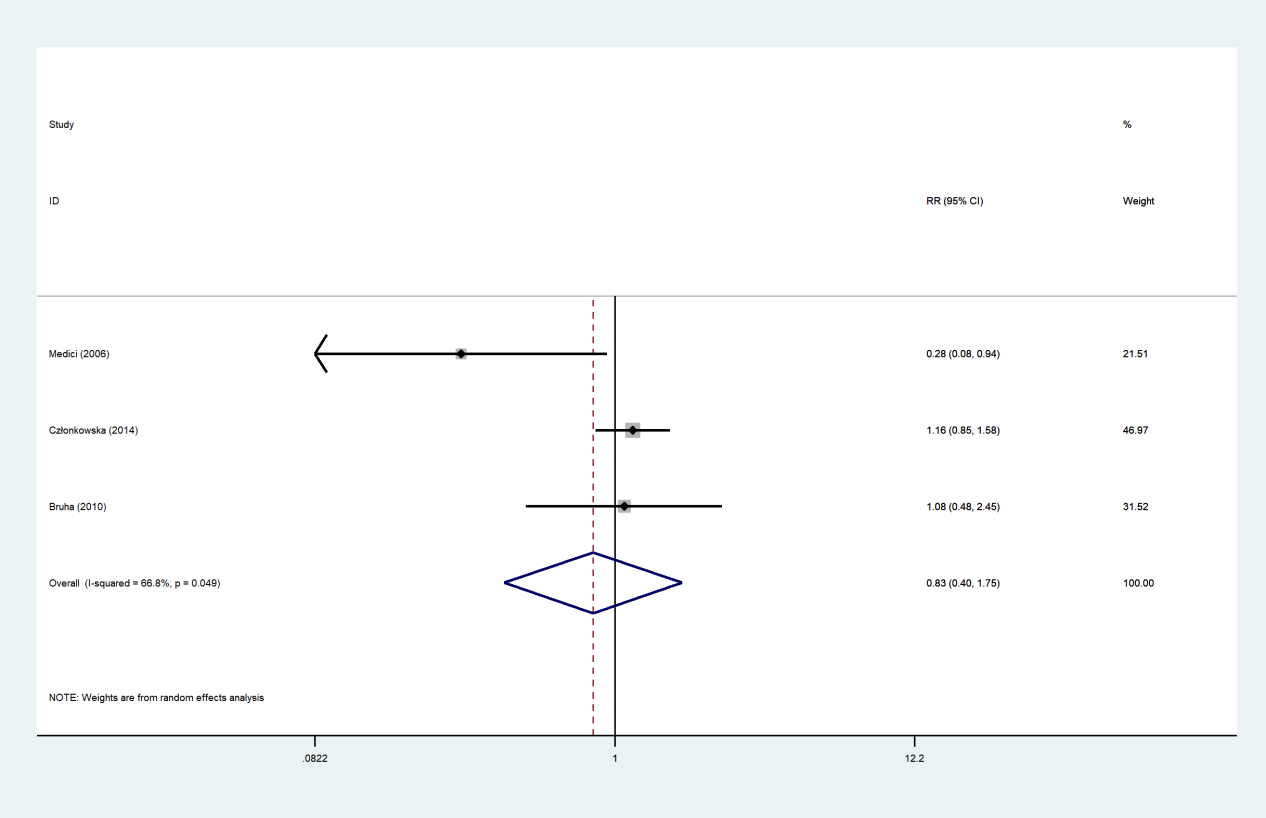
**

**Figure S5. Meta-analysis of treatment effectiveness in neurological WD patients treated with penicillamine compared with zinc salts.**

**
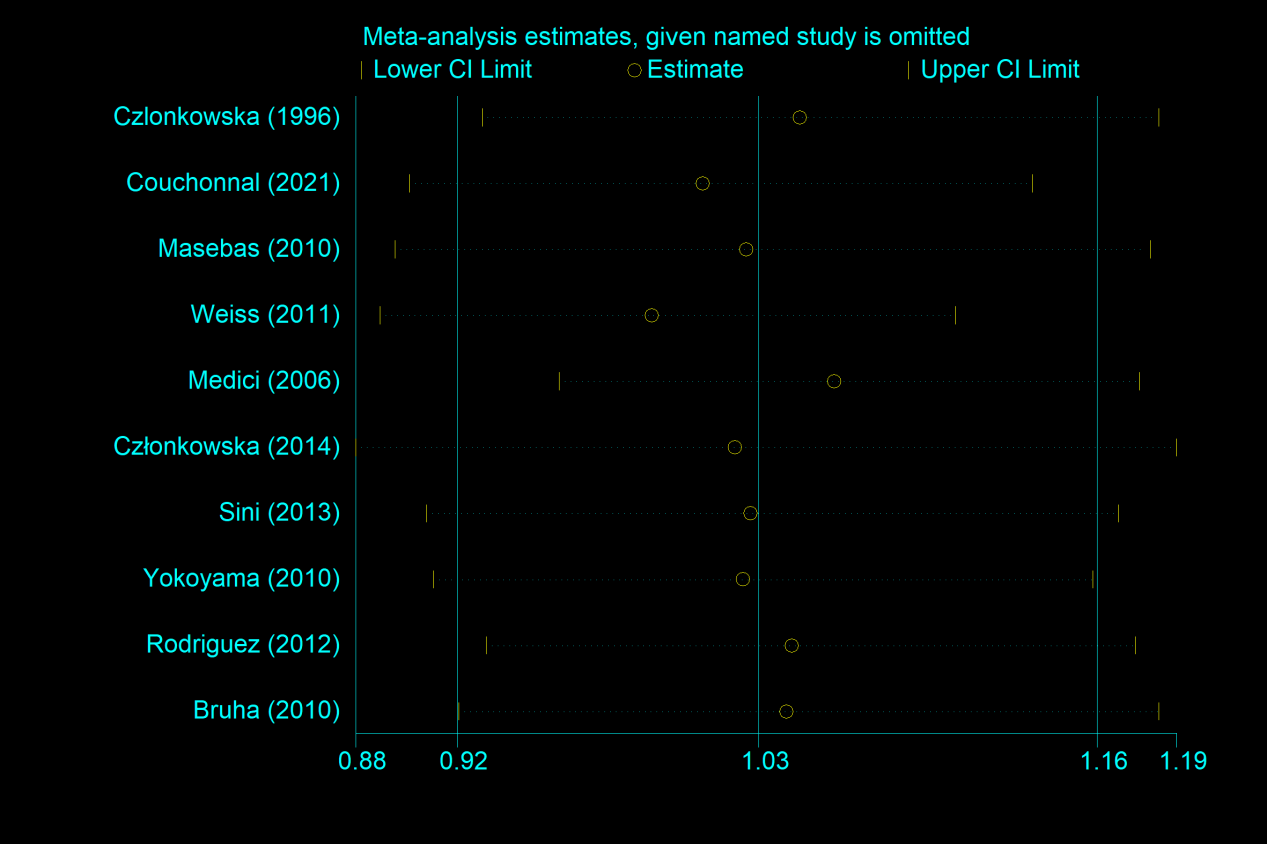
**

**Figure S6. Sensitivity analysis of the treatment effectiveness included studies**

**
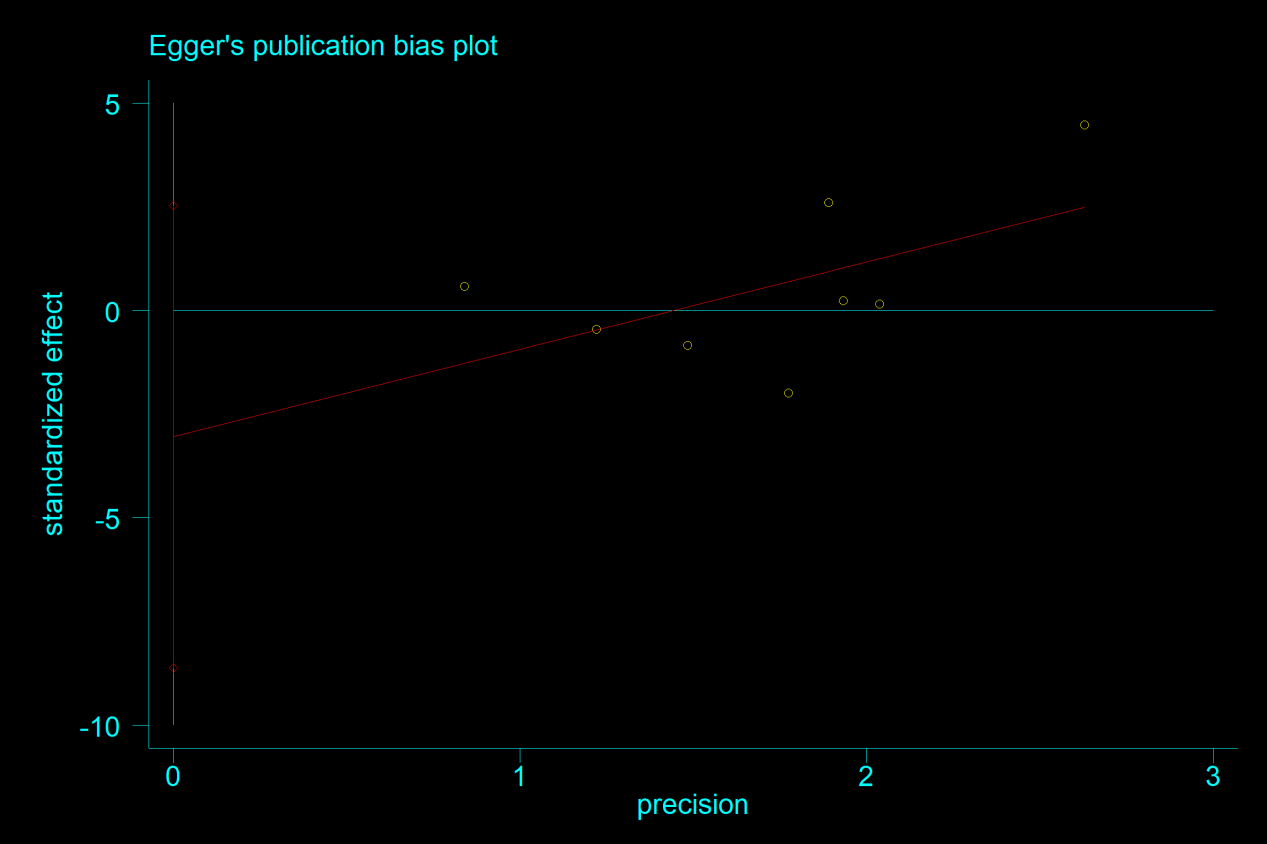
**

**Figure S7. Egger chart of the treatment effectiveness included studies**

**
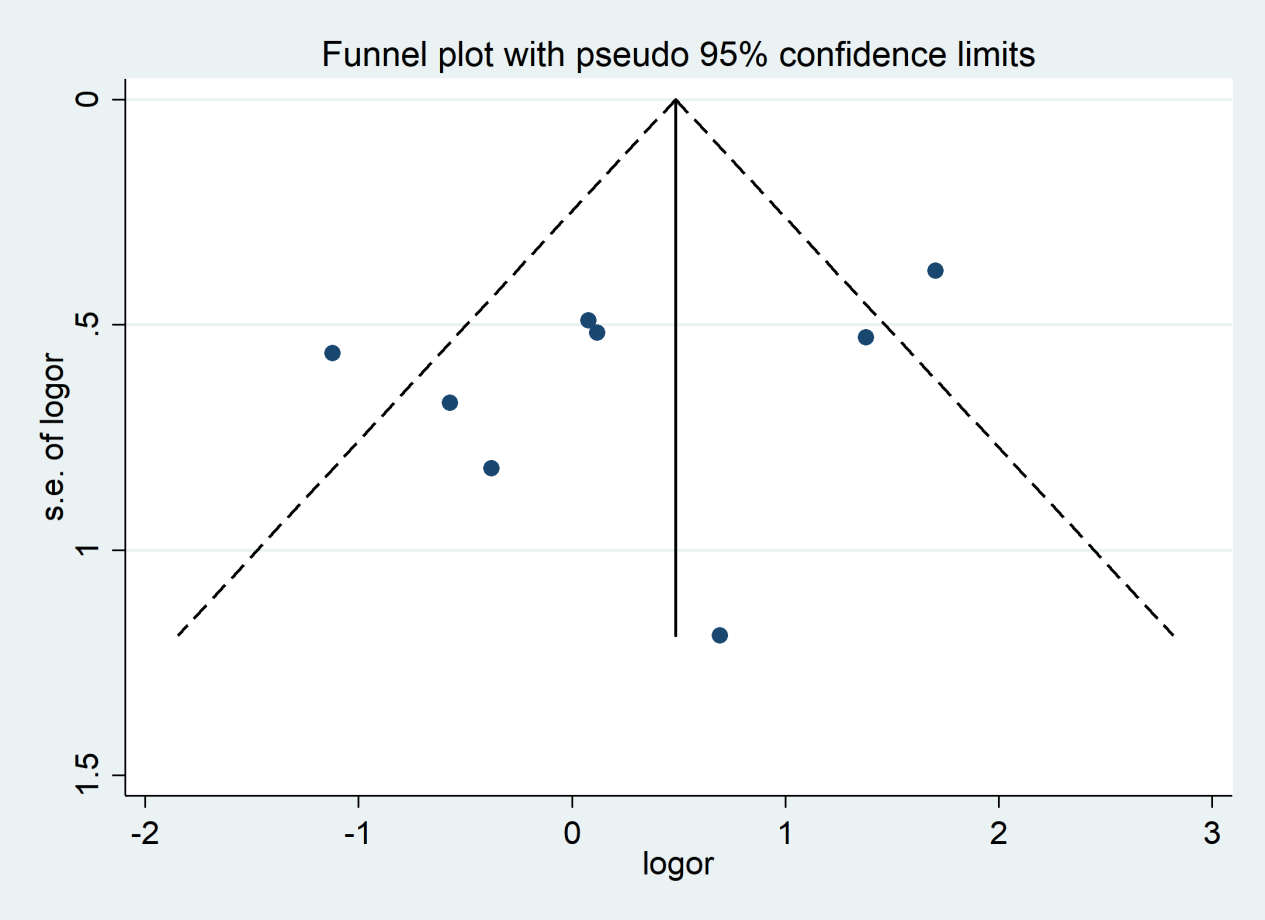
**

**Figure S8. Funnel plot of the treatment effectiveness included studies**

**
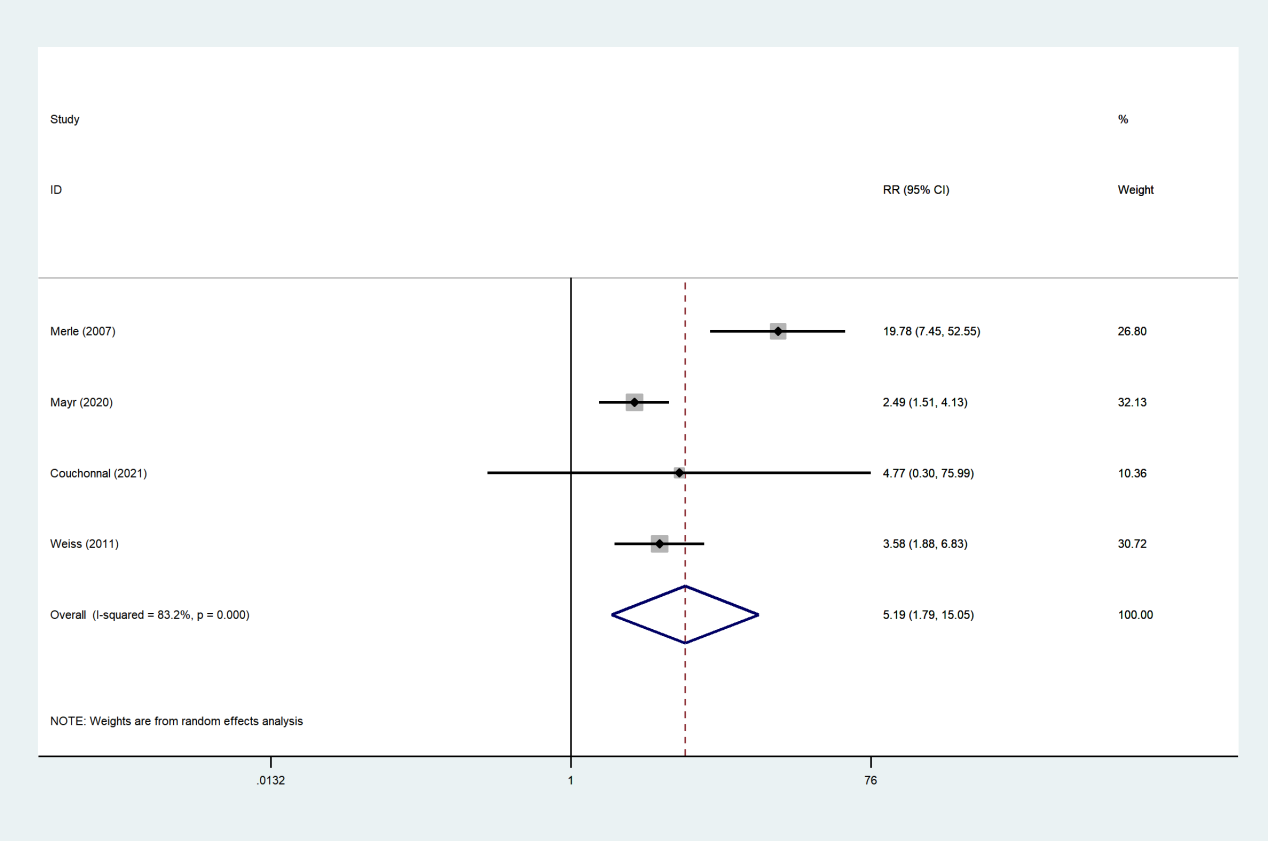
**

**Figure S9. Meta-analysis of adverse effects in WD patients treated with penicillamine compared with trientine**
